# Computational Hemodynamic Analysis for the Diagnosis of Atherosclerotic Changes in Intracranial Aneurysms: A Proof-of-Concept Study Using 3 Cases Harboring Atherosclerotic and Nonatherosclerotic Aneurysms Simultaneously

**DOI:** 10.1155/2016/2386031

**Published:** 2016-09-14

**Authors:** Shin-ichiro Sugiyama, Hidenori Endo, Kuniyasu Niizuma, Toshiki Endo, Kenichi Funamoto, Makoto Ohta, Teiji Tominaga

**Affiliations:** ^1^Department of Neurosurgery, Tohoku University Graduate School of Medicine, Sendai, Japan; ^2^Graduate School of Biomedical Engineering, Tohoku University, Sendai, Japan; ^3^Department of Neuroanesthesia, Kohnan Hospital, Sendai, Japan; ^4^Department of Neurosurgery, Kohnan Hospital, Sendai, Japan; ^5^Frontier Research Institute for Interdisciplinary Sciences, Tohoku University, Sendai, Japan; ^6^Institute of Fluid Science, Tohoku University, Sendai, Japan

## Abstract

This was a proof-of-concept computational fluid dynamics (CFD) study designed to identify atherosclerotic changes in intracranial aneurysms. We selected 3 patients with multiple unruptured aneurysms including at least one with atherosclerotic changes and investigated whether an image-based CFD study could provide useful information for discriminating the atherosclerotic aneurysms. Patient-specific geometries were constructed from three-dimensional data obtained using rotational angiography. Transient simulations were conducted under patient-specific inlet flow rates measured by phase-contrast magnetic resonance velocimetry. In the postanalyses, we calculated time-averaged wall shear stress (WSS), oscillatory shear index, and relative residence time (RRT). The volume of blood flow entering aneurysms through the neck and the mean velocity of blood flow inside aneurysms were examined. We applied the age-of-fluid method to quantitatively assess the residence of blood inside aneurysms. Atherosclerotic changes coincided with regions exposed to disturbed blood flow, as indicated by low WSS and long RRT. Blood entered aneurysms in phase with inlet flow rates. The mean velocities of blood inside atherosclerotic aneurysms were lower than those inside nonatherosclerotic aneurysms. Blood in atherosclerotic aneurysms was older than that in nonatherosclerotic aneurysms, especially near the wall. This proof-of-concept study demonstrated that CFD analysis provided detailed information on the exchange and residence of blood that is useful for the diagnosis of atherosclerotic changes in intracranial aneurysms.

## 1. Introduction

In recent years, computational fluid dynamics (CFD) has attracted attention as a new method to elucidate the hemodynamics of intracranial aneurysms. Progress in CFD enabled hemodynamic simulation of realistic aneurysm geometries with accuracy and reliability [1]. Medical image-based CFD has yielded much new knowledge about the role of hemodynamics in the pathophysiology of intracranial aneurysms [2, 3]. In a guideline for the management of intracranial aneurysms, consideration of hemodynamic characteristics is now recommended when evaluating the risk of aneurysm rupture, in addition to the size and location of the aneurysm and patient age and health status [4–6]. However, little work has been done on the atherosclerotic changes of intracranial aneurysms which is important for predicting aneurysm history or estimating the potential risks of surgical treatments.

The influence of blood flow on the aneurysmal wall is characterized by the hemodynamic wall parameters. Wall shear stress (WSS) is the tangential frictional stress caused by blood flow on the luminal wall [7]. Tateshima et al. published the first study about the influence of hemodynamics on atherosclerotic changes in intracranial aneurysms [8]. Their study using particle image velocimetry suggested that atherosclerotic change occurs in an area exposed to unphysiologically low WSS. Meng et al. hypothesized a biomechanical pathway for atherosclerotic changes in intracranial aneurysms: low and/or oscillatory WSS triggers an inflammatory cell-mediated pathway with pathobiological responses such as proinflammatory change in endothelial cells, infiltration of inflammatory cells, and proliferation of smooth muscle cells [9]. In addition, our previous hemodynamic study of 30 cases with unruptured middle cerebral artery aneurysms suggested that relative residence time (RRT), a hemodynamic wall parameter indicating disturbed blood flow [10], was significantly longer in atherosclerotic aneurysms than nonatherosclerotic aneurysms [11].

This study was intended as a proof of concept for CFD analysis to identify atherosclerotic changes in intracranial aneurysms. We selected 3 patients with multiple aneurysms including at least one with atherosclerotic changes. This patient selection is important because biological environments derived from genetic backgrounds or life habits are equal for all aneurysms in the same host, and only hemodynamic conditions are different.

We conducted a medical image-based CFD study of these 3 cases under patient-specific inlet conditions. In postanalyses, we examined whether RRT could be a discriminant for atherosclerotic aneurysms and further investigated the exchange and residence of blood inside intracranial aneurysms. Using the age-of-fluid concept from the field of engineering [12], we estimated the realistic residence time of blood and the distribution of the age of blood inside aneurysms. This study demonstrates that the age-of-fluid method provides information on the exchange and residence of blood that is useful for the diagnosis of atherosclerotic changes in intracranial aneurysms.

## 2. Materials and Methods

### 2.1. Patient Population

We selected 3 patients with multiple unruptured aneurysms including at least one with atherosclerotic changes in each patient. We distinguished atherosclerotic changes on aneurysmal wall by remarkable yellow lipid deposition at surgery. Clinical information was collected from medical records. The study was approved by the institutional review board of Kohnan Hospital.

### 2.2. Measurement of Flow Rates in Parent Arteries

Quantitative magnetic resonance (MR) velocimetry was performed with a 3-T MR image scanner (Signa HDxt; GE Healthcare Japan, Tokyo, Japan) before surgical treatment [13]. The protocol uses standard cranial three-dimensional (3D) time-of-flight MR angiography to select slice orientation for arterial blood flow measurements. The optimal perpendicular scan plane was determined from the acquired time-of-flight images. The coordinates obtained specified the position of an oblique fast two-dimensional phase-contrast sequence that was then performed on the basis of these coordinates using a peripheral gated two-dimensional phase-contrast sequence with the following imaging parameters: repetition time/echo time/number of excitations, 25 milliseconds/5.4 milliseconds/1; field of view, 160 · 160 mm; matrix, 512 · 512; voxel size, 0.3 · 0.3 mm; velocity encoding, 100 cm/s; imaging time, about 5 minutes; direction, transaxial; peripheral gated with electrocardiogram (ECG); and phases, 30. The acquired phase-contrast images were transferred to the workstation for flow quantification with dedicated software (CV Flow; GE Healthcare Japan). A region of interest was placed semiautomatically on the phase-contrast images over a cardiac cycle. The velocities at all of the pixels inside the vessel border were integrated to calculate the flow in milliliters per second, and these values were used to obtain the quantitative waveform over the cardiac cycle.

### 2.3. CFD Modeling

#### 2.3.1. Geometrical Model Construction

**Figure 1:**
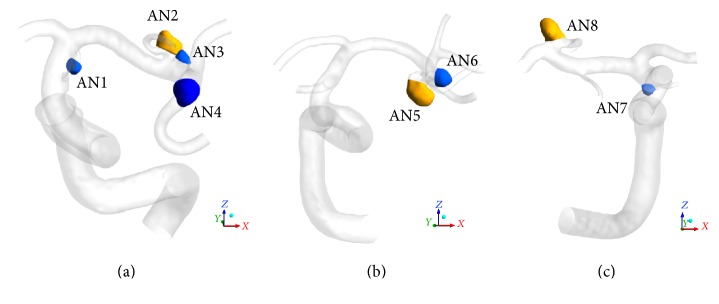
Computational domains and segmentation of aneurysms in Case 1 (a), Case 2 (b), and Case 3 (c). Aneurysms with atherosclerotic changes are colored yellow (AN2, AN5, and AN8) and others without atherosclerotic changes blue.

Conventional digital subtraction and 3D rotational angiography were performed by standard transfemoral catheterization with biplane units (Innova 3131 or IGES 630; GE Healthcare Japan, Tokyo, Japan). These images were obtained during a 6-second injection of a contrast agent and 200 rotations with imaging at 30 frames per second for 5 seconds. The 150 projection images were reconstructed into a three-dimensional data set of 512 · 512 · 512 isotropic voxels covering a field of view of 200 mm in all 3 directions. The 3D data set obtained from rotational angiography was exported to a personal computer to form a 3D isosurface model of the aneurysms. A 3D surface was extracted with the marching cube method using open source software (Vascular Modeling Tool Kit; VMTK (http://www.vmtk.org/)) [14]. The segmented 3D surface was then processed with commercial software (Magics RP 13.1; Materialise, Leuven, Belgium) to clear small branches from the regions of interest and to make planes for inlets and outlets. Inlets were set in the cervical portion of the ipsilateral internal carotid artery (ICA) to ensure proper length for calculation. The results of geometrical modeling were validated by two-dimensional conventional angiograms in the anteroposterior and lateral views and intraoperative videograms of vessels and aneurysms exposed during surgery. The neck plane for each aneurysm was manually positioned to measure the volume of blood flow into the aneurysm through the neck (Figure 1).

#### 2.3.2. Numerical Simulations

All models used in the computational study were meshed with the use of commercial software (ICEM CFD; ANSYS Inc., Lebanon, NH, USA) to create tetrahedral elements with 5 layers of finer prism elements in the boundary, resulting in approximately 1.5 million elements. A finite-volume method package, ANSYS 14.5 (ANSYS Inc.), was used to solve the governing equations: 3D unsteady Navier-Stokes equations and equation of continuity. As for the computational algorithm, the SIMPLE method was used. The Green-Gauss node-based gradient method was applied for the differential and gradient calculations, and the PRESTO! method and QUICK method were used for pressure completion and convection discretization, respectively. Pulsatile flow rates of ICAs measured by MR velocimetry were prescribed at the inlet boundary. The diffusion fluxes in the direction normal to the inlet plane were assumed to be zero, and normal gradients were neglected. The proportion of flow rates for outlets was determined according to the principle of minimum work (Murray's law).

Regarding the viscosity of fluid, we tested two settings. One followed the conventions for CFD in large vessels [2]. Blood was modeled as an incompressible Newtonian fluid with a density of 1,050 kg/m^3^ and a viscosity of 0.0035 kg/ms. The other was carried out for non-Newtonian flow. The non-Newtonian blood viscosity was modeled with the Herschel-Bulkley formula, which accounts for yield stress and shear thinning properties of blood [15, 16]:

(1) $$\begin{array}{c} \mu =k{\dot{\gamma }}^{\left(n-1\right)}+\frac{\tau_{0}}{\dot{\gamma }}, \end{array}$$

where $\dot{\gamma }$ is strain rate and *τ*_0_ is yield stress. The experimental values reported by Kim were used for coefficients *k* and *n* [17]. The Herschel-Bulkley model is popular in recent studies because it captures shear thinning and fits over a range of shear rates [15, 16, 18–20].

A rigid wall no-slip boundary condition was implemented at the vessel walls. Three pulsatile cycles were simulated to ensure that numeric stability was reached, and the results from the third cycle were used for analysis.

### 2.4. Hemodynamic Wall Parameters

Three hemodynamic wall parameters were calculated: RRT, time-averaged WSS, and oscillatory shear index (OSI) [7, 10]. Supplemental Content 1 (in Supplementary Material available online at http://dx.doi.org/10.1155/2016/2386031) gives a detailed description of WSS, OSI, and RRT. The maximum RRT, minimum WSS, and maximum OSI of each aneurysm were calculated.

### 2.5. The Proportion of Inflow Volume to Aneurysm Volume

We measured the volume of aneurysms (aneurysm volume (mm^3^)) from the data of 3D geometry. We also calculated the volume of blood flow entering aneurysms through the neck (inflow volume (mm^3^/cycle)) from the numerical results. We calculated the proportion of the inflow volume to the aneurysm volume as exchange rate (1/cycle) to assess the exchange of blood inside aneurysms, because poor exchange of blood results in long residence time. We also calculated the mean blood velocity inside each aneurysm.

### 2.6. The Age-of-Blood Method

In the field of CFD, Haimes first proposed the concept of residence time as the amount of time the fluid has been in (or resident within) the domain [12]. This concept is used as the “age of air” in heating, ventilating, and air conditioning (HVAC) design or as the “age of fluid” in oceanic studies in order to estimate the local ventilation or fluid exchange by CFD where real measurement is impossible [21].

The age of fluid was defined as the Eulerian transposition of the Lagrangian age [12, 21]. The age of a fluid parcel is the time elapsed since the parcel left the inlet in which its age is prescribed to be zero. When we consider a fluid parcel following a path described by its position vector **r**(*t*) at time *t*, **r**(*t*) is related to the velocity **u** between two instants *t*_0_ and *t*.

(2) $$\begin{array}{c} r\left(\;t\;\right)=r\left(\;t_{0}\;\right)+\overset{t}{\underset{t_{0}}{\int }}u\left[\;s,r\left(\;s\;\right)\;\right]ds \end{array}$$

The age *a*[*t*, **r**(*t*)] of the fluid parcel at position **r** at time *t* is given by

(3) $$\begin{array}{c} a\left[\;t,r\left(\;t\;\right)\;\right]\equiv t-t_{0}+a\left[\;t_{0},r\left(\;t_{0}\;\right)\;\right]. \end{array}$$

We can assume that *t*_0_ is the time when the fluid parcel left the inlet where the age is prescribed to be zero: *a*[*t*_0_, **r**(*t*_0_)] ≡ 0. Therefore, we obtain the fundamental expression

(4) $$\begin{array}{c} r\left(\;t\;\right)-r\left(\;t_{0}\;\right)=\overset{t}{\underset{t-a\left[t,r\left(t\right)\right]}{\int }}u\left[\;s,r\left(\;s\;\right)\;\right]ds \end{array}$$

which means the age of a fluid parcel is the time elapsed along its path.

The derivative of (3) along the path and its Eulerian equivalent are as follows:

(5) DDtat,rt=1

(6) ∂∂ta+∇·ua=1.

We applied the “age-of-fluid” concept to the residence time of blood. Therefore, we call it the “age-of-blood” method, and the obtained value is the “age of blood” or “blood age.” In our preliminary study using simple geometries, we confirmed that we could assess the residence time of blood using the age-of-blood method [22]. For the assessment of blood age in this study, 5 additional cardiac cycles were simulated according to (6) after 3 cardiac cycles to obtain flow fields.

We calculated the spatially averaged value and the maximum value of “blood age” inside the aneurysmal domain over 5 cardiac cycles.

## 3. Results

### 3.1. Patient Information

#### 3.1.1. Case 1

**Figure 2:**
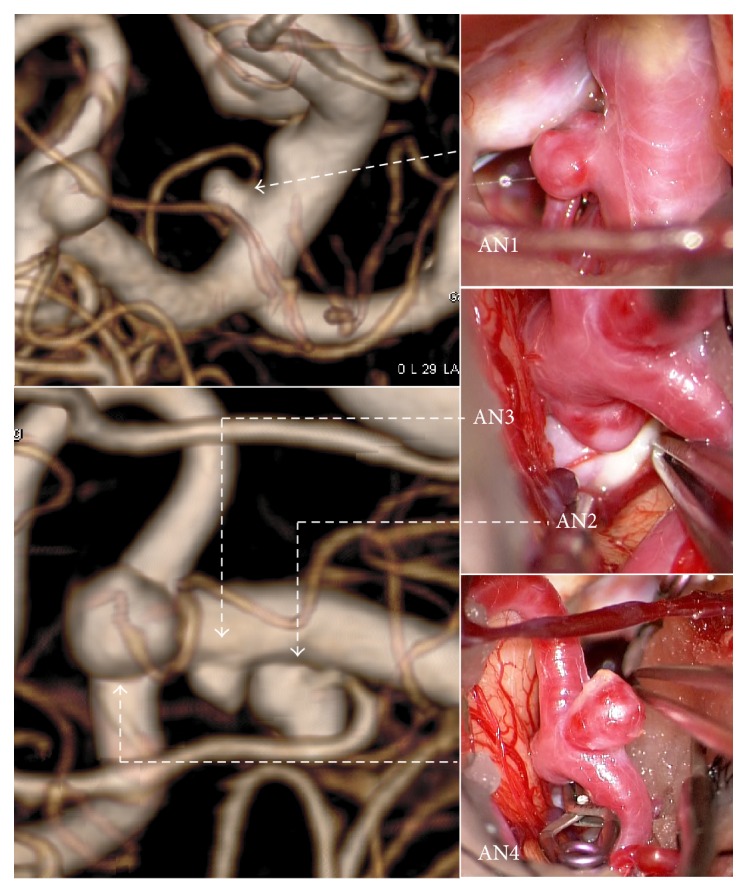
Images of rotational angiography and intraoperative photographs showing the locations and appearances of aneurysms in Case 1. Atherosclerotic changes were observed in AN2.

A 62-year-old female presented with 7 unruptured aneurysms (Table 1). Four were on the left side of the anterior circulation (Figures 1 and 2): one at the bifurcation of the left internal carotid artery (ICA) and anterior choroidal artery (AN1), 2 adjacent ones in the horizontal part of the left middle cerebral artery (MCA) (AN2 and AN3), and one at the bifurcation of the left MCA (AN4). The remaining 3 aneurysms included one at the clinoid segment of the right ICA, one at the bifurcation of the right MCA, and one at the bifurcation of the basilar artery and right superior cerebellar artery. Clipping surgery was planned for 4 aneurysms in the left anterior circulation. At surgery, the left M1 aneurysm (AN2) had thick wall with yellow lipid deposition over the dome, whereas the other 3 aneurysms (AN1, AN3, and AN4) had a reddish dome with a white patch (Figure 2). It was difficult to perform neck clipping for the left M1 aneurysm due to prominent atherosclerotic changes. Three reddish aneurysms were all clipped, but wrapping was performed for the atherosclerotic aneurysm.

#### 3.1.2. Case 2

**Figure 3:**
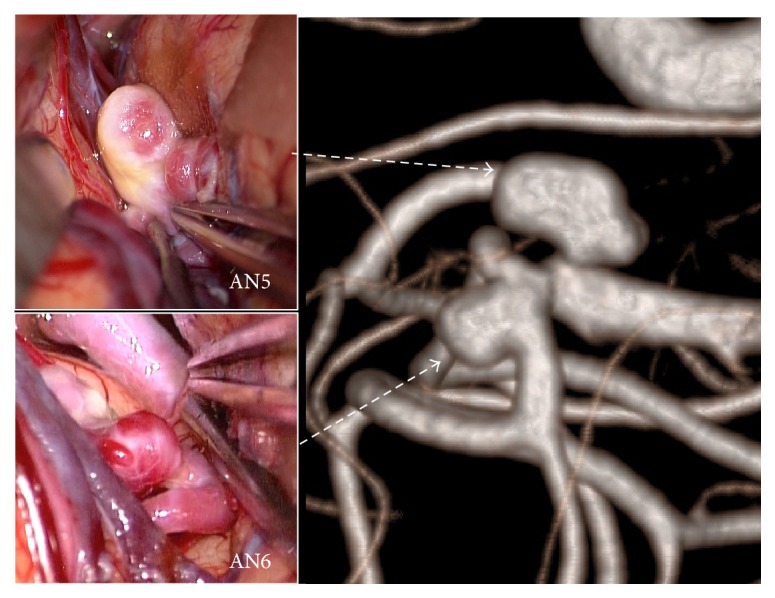
Images of rotational angiography and intraoperative photographs showing the locations and appearances of aneurysms in Case 2. Atherosclerotic changes were observed in AN5.

A 65-year-old female presented with 3 unruptured aneurysms (Table 1). Two were in the left MCA (Figures 1 and 3): one at the bifurcation of the inferior trunk and the middle trunk (angular artery) (AN5) and one at the bifurcation of the middle trunk (angular artery) and the superior trunk (AN6). Another aneurysm was in the right MCA. Clipping surgery was planned for 2 aneurysms in the left MCA. At surgery, the proximal aneurysm (AN5) had a heterogeneous dome with atherosclerotic changes, whereas the distal one (AN6) had a reddish dome with a thin bleb (Figure 3). Neck clipping was difficult for the proximal aneurysm due to atherosclerotic changes including the neck, and incomplete clipping was performed at the dome.

#### 3.1.3. Case 3

**Figure 4:**
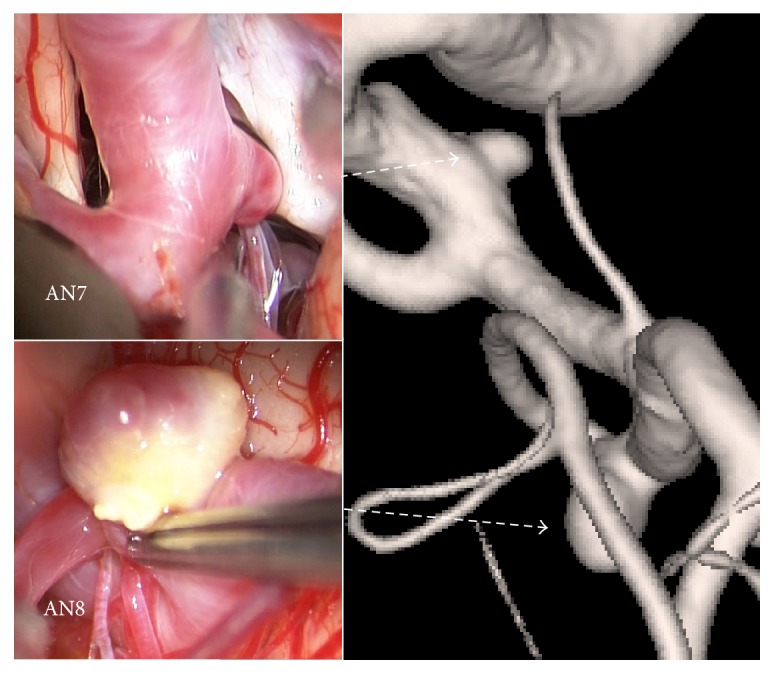
Images of rotational angiography and intraoperative photographs showing the locations and appearances of aneurysms in Case 3. Atherosclerotic changes were observed in AN8.

A 61-year-old male presented with 2 unruptured aneurysms in the right anterior circulation (Table 1, Figures 1 and 4): one at the bifurcation of the left ICA and anterior choroidal artery (AN7) and one at the bifurcation of the MCA (AN8). Clipping surgery was planned for the 2 aneurysms. At surgery, the MCA aneurysm (AN8) had a heterogeneous dome with atherosclerotic changes, whereas the ICA-anterior choroidal artery aneurysm had a reddish dome without atherosclerotic changes (Figure 4). In this case, we could perform neck clipping for both aneurysms.

### 3.2. Results of Computational Hemodynamic Simulation

We conducted hemodynamic simulations under 2 boundary conditions for the blood viscosity. We found that Herschel-Bulkley models for non-Newtonian flow generally showed little deviation from the Newtonian viscosity for aneurysms without atherosclerotic changes (AN1, AN3, AN4, AN6, and AN8), but not for aneurysms with atherosclerotic changes (AN2, AN5, and AN8), where they predicted much higher viscosity. Supplemental Content 2 demonstrates the distribution of blood viscosity in each aneurysm. The latter deviation was due to the decrease of blood velocity, which may prolong the residence time in specific aneurysms. Therefore, the numerical results using Herschel-Bulkley models are demonstrated hereafter.

#### 3.2.1. Case 1

**Figure 5:**
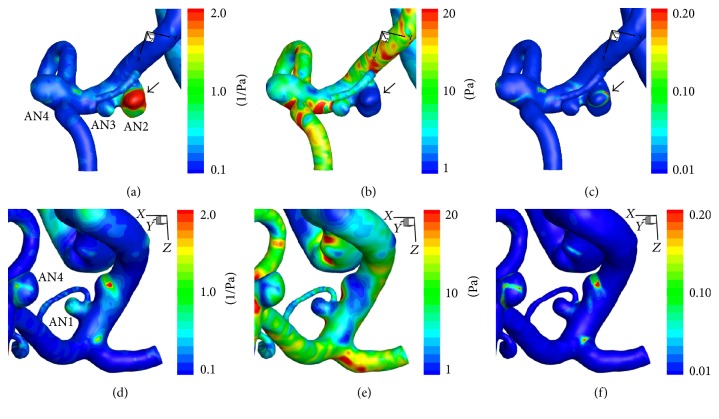
Contour maps of RRT ((a) and (d)), time-averaged WSS ((b) and (e)), and OSI ((c) and (f)) in Case 1. The aneurysm with atherosclerotic changes (AN2) presented with long RRT and low WSS. Black arrows indicated the point with the longest RRT (117.3 (1/Pa)), the lowest WSS (0.02 (Pa)), and the highest OSI (0.36) in AN2. See also Table 2. RRT: relative residence time, WSS: wall shear stress, and OSI: oscillatory shear index.

*Hemodynamic Wall Parameters*. The maximum RRT, the minimum WSS, and the maximum OSI in the left MCA aneurysm with atherosclerotic changes (AN2) were longer, lower, and higher than those in the other 3 aneurysms with reddish domes (Table 2). In the contour maps of these 3 parameters, AN2 presented with long RRT and low WSS (Figures 5(a)–5(f)).

**Figure 6:**
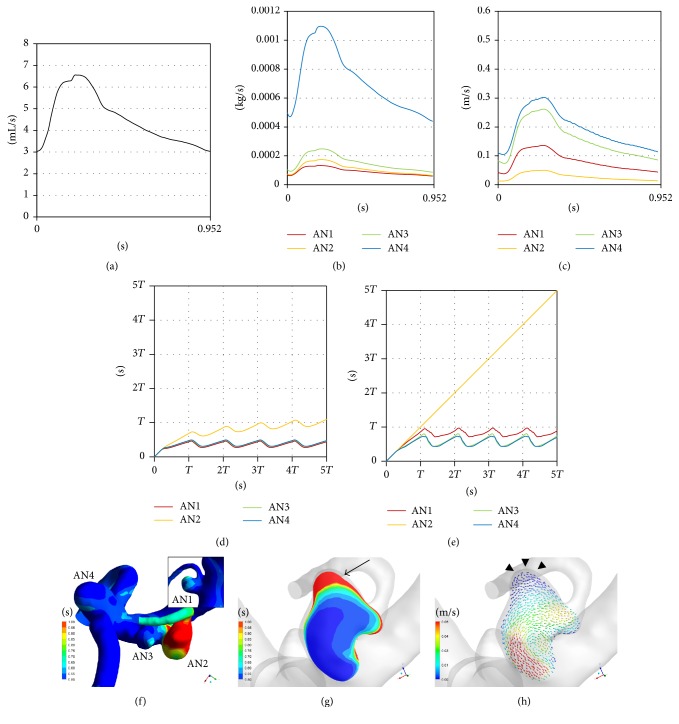
((a)–(e)) Graphs showing the results of computational hemodynamic analysis in Case 1. The graph of the atherosclerotic aneurysm (AN2) is colored yellow. (a) Pulsatile flow rates of the internal carotid artery measured by phase-contrast MR (volume, 271.7 (mL/min); heart rate, 63 (bpm); heart beat duration (*T*), 0.952 (s)). (b) Volume of inflow. (c) Mean flow velocity inside aneurysms. (d) The spatially averaged value of blood age inside the aneurysms. (e) The maximum values of blood age inside the aneurysms. Note that the maximum age inside AN2 increased linearly, and the inclination was unity over 5 cardiac cycles. (f) Contour maps of blood age (*t* = 5*T*). (g) A cutting plane of blood age in AN2 (*t* = 5*T*). (h) Velocity vectors on the cutting plane (*t* = 5*T*). Old blood was seen near the wall (a black arrow in (g)) due to recirculatory flow (arrowheads in (h)). MR: magnetic resonance.

*Hemodynamics inside Aneurysms*. Blood flow entered aneurysms periodically (Figures 6(a)–6(c)). The volume of inflow in AN2 was larger than that in AN1 and smaller than that in AN3 and AN4 (Table 3 and Figure 6(b)). However, the proportion of inflow volume to aneurysm volume (exchange rate) was smaller in AN2 than in AN1 (Table 3). In addition, the mean flow velocity inside AN2 was smaller than in the other 3 aneurysms including AN1 (Figure 6(c)).

*Age of Blood inside Aneurysms*. Residence time of blood inside an aneurysm was evaluated using the age-of-blood method. The mean and maximum age of blood inside each aneurysm changed periodically and were opposite in phase with the inlet flow rates, the inflow volume, and the pulsatile changes of mean flow velocity (Figures 6(a)–6(e)). The maximum age of blood inside 3 aneurysms with reddish walls (AN1, AN3, and AN4) changed periodically with the mean age of blood (Figures 6(d) and 6(e)). On the other hand, the maximum age inside AN2 increased linearly, and the inclination was unity (Figure 6(e)), which means that there is a region with extremely slow recirculation. In AN2, the distribution of the age of blood was similar to the distribution of RRT (Figures 5(a) and 6(f)). In the cutting plane, the old blood coincided with recirculatory flow with slow velocity (Figures 6(g) and 6(h)).

#### 3.2.2. Cases 2 and 3

**Figure 7:**
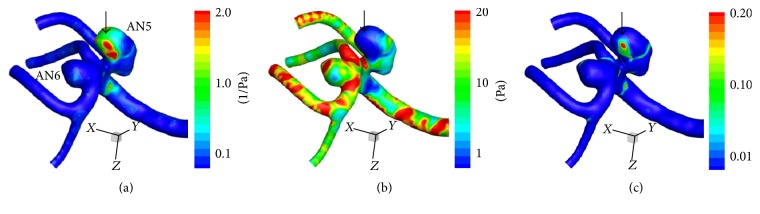
Contour maps of RRT (a), time-averaged WSS (b), and OSI (c) in Case 2. The aneurysm with atherosclerotic changes (AN5) presented with long RRT and low WSS. Black arrows indicate the point with the longest RRT (11.6 (1/Pa)), the lowest WSS (0.2 (Pa)), and the highest OSI (0.32) in AN5. See also Table 2. RRT: relative residence time, WSS: wall shear stress, and OSI: oscillatory shear index.

**Figure 8:**
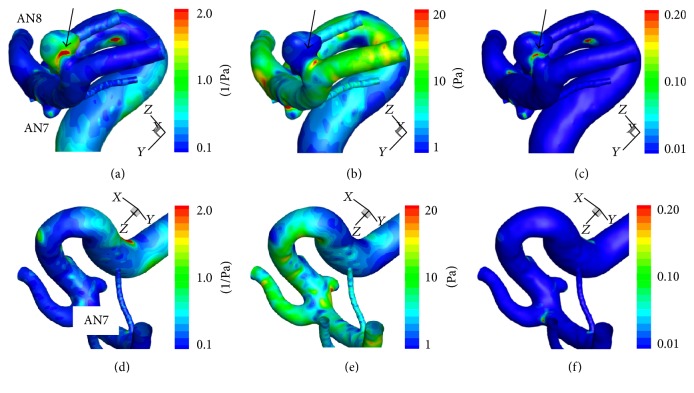
Contour maps of RRT ((a) and (d)), time-averaged WSS ((b) and (e)), and OSI ((c) and (f)) in Case 3. The graph of the atherosclerotic aneurysm (AN8) is colored yellow. The aneurysm with atherosclerotic changes (AN8) presented with long RRT and low WSS. Black arrows indicate the point with the longest RRT (5.3 (1/Pa)), the lowest WSS (0.2 (Pa)), and the highest OSI (0.29) in AN8. See also Table 2. RRT: relative residence time, WSS: wall shear stress, and OSI: oscillatory shear index.

*Hemodynamic Wall Parameters*. The maximum RRT, the minimum WSS, and the maximum OSI in the atherosclerotic aneurysms (AN5 in Case 2 and AN8 in Case 3) were longer, lower, and higher than the other nonatherosclerotic ones (AN6 in Case 2 and AN7 in Case 3) (Table 2). In the contour maps of these 3 parameters, two atherosclerotic aneurysms (AN5 and AN8) presented with long RRT and low WSS (Figures 7(a)–7(c) and 8(a)–8(f)). In visual inspection of the intraoperative video recording and the contour maps of RRT, the atherosclerotic changes in AN5 and AN8 coincided with the area with long RRT (Figures 3, 7(a), 4, and 8(a), resp.).

**Figure 9:**
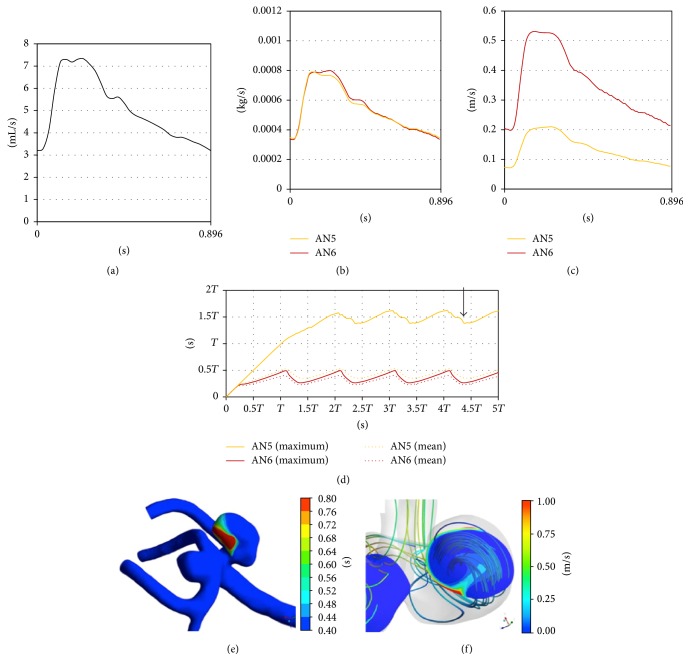
((a)–(d)) Graphs showing the results of computational hemodynamic analysis in Case 2. The graph of the atherosclerotic aneurysm (AN5) is colored yellow. (a) Pulsatile flow rates of the internal carotid artery (volume, 305.2 (mL/min); heart rate, 67 (bpm); heart beat duration (*T*), 0.896 (s)). (b) Volume of inflow. (c) Mean flow velocity inside aneurysms. (d) The spatially averaged value and the maximum value of blood age over 5 cardiac cycles. ((e) and (f)) A surface (e) and a cutting plane (f) of the contour of blood age in AN5 at the time point shown in (d) (a black arrow). Streamlines are also shown in (f) to demonstrate vortex flow inside the aneurysm. Note that old blood was seen at the center of the vortex. WSS: wall shear stress.

**Figure 10:**
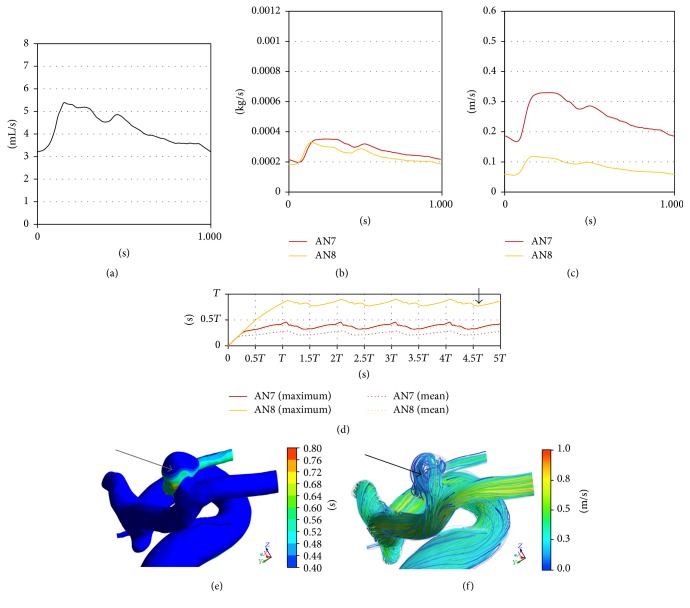
((a)–(d)) Graphs showing the results of computational hemodynamic analysis in Case 3. (a) Pulsatile flow rates of the internal carotid artery (volume, 252.7 (mL/min); heart rate, 60 (bpm); heart beat duration (*T*), 1.000 (s)). (b) Volume of inflow. (c) Mean velocity inside aneurysms. (d) The spatially averaged value and the maximum value of blood age over 5 cardiac cycles. ((e) and (f)) A contour map of blood age (e) and streamlines (f) in AN8 at the time point shown in (d) (a black arrow). Note that old blood was seen around the center of the vortex indicated by streamlines (a black arrow in (f)). WSS: wall shear stress.

*Hemodynamics inside Aneurysms*. Blood flow entered aneurysms periodically (Figures 9(a)–9(c) and 10(a)–10(c)). The inflow volumes of atherosclerotic aneurysms (AN5 and AN8) were almost equal to the other nonatherosclerotic ones (AN6 and AN7) (Table 3, Figures 9(b) and 10(b)). However, the mean flow velocity and the exchange rate were smaller in the atherosclerotic aneurysms than in the other nonatherosclerotic aneurysms (Figures 9(c) and 10(c) and Table 3).

*Age of Blood inside Aneurysms*. The mean age of blood inside each aneurysm was opposite in phase with the inlet flow rates, the inflow volume, and the mean flow velocity (Figures 9(d) and 10(d)). The maximum ages of blood in the atherosclerotic aneurysms (AN5 and AN8) also changed periodically but fluctuated at a higher value than those in the other nonatherosclerotic aneurysms (AN6 and AN7).

In AN5, the distribution of the age of blood was similar to the distribution of RRT (Figures 7(a) and 9(e)). In the cutting plane, the old blood was seen in the center of vortex inside AN5 (Figure 9(f)).

In AN8, the distribution of the age of blood was similar to the distribution of RRT (Figures 8(a) and 10(e)). The old blood was seen around the unstable vortex inside the aneurysm (Figure 10(f)).

## 4. Discussion

This computational study revealed 2 major hemodynamic differences between atherosclerotic and nonatherosclerotic aneurysms. The first concerns disturbed blood flow. Aneurysms with atherosclerotic changes were exposed to disturbed blood flow, indicated by long RRT, as reported previously [11]. In particular, the aneurysm in Case 1 with prominent atherosclerotic changes (AN2) showed remarkable prolongation of RRT (117.3 (1/Pa)). On the other hand, aneurysms without atherosclerotic changes were exposed to more physiological conditions (RRT < 2.0 (1/Pa)).

The second point concerns the residence time of blood inside aneurysms. The age-of-blood method revealed that the mean and maximum age of blood inside atherosclerotic aneurysms were higher than those inside nonatherosclerotic aneurysms, and the old blood inside atherosclerotic aneurysms localized at the area exposed to the disturbed flow, as indicated by long RRT (and low WSS). In particular, the maximum age inside AN2 increased linearly, and the inclination was unity, which means that there is a region with extremely slow recirculation. The proportion of inflow volume to aneurysm volume was smaller in atherosclerotic than in nonatherosclerotic aneurysms, and the mean blood flow velocity was slower in atherosclerotic than in nonatherosclerotic aneurysms. We speculate that the localized disturbed blood flow that yields low WSS and long blood residence occurs when the volume of aneurysm increases due to aneurysm growth, and the mean blood velocity decreases to a certain threshold.

Himburg et al. proposed RRT as an indicator for the existence of disturbed blood flow near the vascular wall [10]. According to the mathematical definition, the RRT is inversely proportional to WSS magnitude and is proportional to OSI, which means relative stagnation of blood near the wall [10]. In a previous study of 30 cases with unruptured aneurysms, statistical analysis showed that the prolongation of RRT as well as male sex was independent significant indicator of atherosclerotic changes in intracranial aneurysms [11]. In the current study, we investigated cases with multiple atherosclerotic and nonatherosclerotic aneurysms and confirmed that the prolongation of both age of blood and RRT was observed in atherosclerotic but not nonatherosclerotic aneurysms. We emphasized the importance of the fact that biological environments surrounding aneurysms are equal in the same host and only hemodynamic conditions are different. However, we should note the difference of elapsed time after the initiation of aneurysms.

In CFD simulations, typical simplifications are made in model generation, the boundary condition settings related to wall compliance and outflow conditions, and the numerical technique used to solve the governing equations. These effects are considered to be fairly small if the vascular geometry is correctly modeled [23]. To measure the volume of inflow or the volume of aneurysm, we positioned the neck plane manually. To obtain more objective values, computational automatic techniques should be utilized for identifying the neck plane of intracranial aneurysms [24].

An important limitation is that we only investigated the appearance of aneurysms at surgery and did not examine the specimen. Though atherosclerotic changes in intracranial aneurysms may be promoted by the same inflammatory pathway as atherosclerosis in large arteries, they seem to have a different etiology. For instance, vulnerable plaque or hemorrhage inside the plaque is frequently seen in atherosclerosis in large arteries but not in intracranial aneurysms. We should make an effort to examine intracranial aneurysm specimens using pathological and biochemical methods with special attention to the inflammatory response that may lead to atherosclerotic changes, although there is little opportunity to obtain a specimen of the aneurysm wall while maintaining complete safety after clipping.

This proof-of-concept study demonstrates that computational hemodynamic analysis using the age-of-blood method enables detailed characterization of stagnant blood flow in intracranial aneurysms. Low WSS causes endothelial dysfunction. Long residence of blood enables inflammatory cells or atherosclerotic particles to infiltrate aneurysm walls. Both may promote atherosclerotic changes in intracranial aneurysms. However, further studies with a larger cohort and/or a prospective design with sufficient statistical power should be conducted to enable application of computational hemodynamic analysis to the diagnosis of atherosclerotic aneurysms that are difficult to clip and should instead receive endovascular treatment.

## Supplementary Material

Content 1, supplemental methods describing the difinition of three hemodynamic wall parameters. Content 2, supplemental results showing the difference of blood viscosity distribution under two rheology models.

## Figures and Tables

**Table 1:** Clinical information in 3 patients with multiple intracranial aneurysms.

Case number	Age Sex	Number of aneurysms	Location of aneurysms	Smoking	Other risks of atherosclerosis
1	62 F	7	Left ICA (1), left MCA (3), Right ICA (1), right MCA (1), BA (1)	No	Hypertension

2	65 F	3	Left MCA (2), right MCA (1)	No	Hypertension Dyslipidemia

3	61 M	2	Right ICA (1), right MCA (1)	Yes	Cerebral infarction

**Table 2:** Atherosclerotic changes and hemodynamic wall parameters in 3 cases.

Case number	Aneurysm number	Atherosclerotic changes	Hemodynamic wall parameters		
			Maximum value of RRT (1/Pa)	Minimum value of WSS (Pa)	Maximum value of OSI
1	AN1	No	4.09	0.49	0.31
	AN2	Yes	117.30	0.02	0.36
	AN3	No	0.93	1.53	0.15
	AN4	No	1.79	0.98	0.29

2	AN5	Yes	11.57	0.20	0.32
	AN6	No	0.44	2.57	0.26

3	AN7	No	1.37	1.01	0.28
	AN8	Yes	5.32	0.20	0.29

RRT: relative residence time, WSS: wall shear stress, and OSI: oscillatory shear index.

**Table 3:** Aneurysm volume, inflow volume, and exchange rate.

Case number	Aneurysm number	Aneurysm volume (*a*) (mm^3^)	Inflow volume (*b*) (mm^3^/cycle)	Exchange rate (*b*/*a*) (1/cycle)
1	AN1	4.94	83.93	17.0
	AN2	18.3	100.0	5.46
	AN3	3.03	139.3	46.0
	AN4	23.6	651.8	27.6

2	AN5	38.6	467.2	12.1
	AN6	15.8	461.3	29.2

3	AN7	4.38	266.6	60.1
	AN8	31.3	234.0	7.48
