# Supplementary material for: Computational Hemodynamic Analysis for the Diagnosis of Atherosclerotic Changes in Intracranial Aneurysms: A Proof-of-Concept Study Using 3 Cases Harboring Atherosclerotic and Nonatherosclerotic Aneurysms Simultaneously
Source: Comput Math Methods Med. 2016 Sep 14;2016:2386031. doi: 10.1155/2016/2386031 (PMC5040129; doi:10.1155/2016/2386031)
Supplement: Supplementary file 1 — Content 1, supplemental methods describing the difinition of three hemodynamic wall parameters. Content 2, supplemental results showing the difference of blood viscosity distribution under two rheology models. [file 2386031.f1.pdf]

Supplemental Content 1

Hemodynamic wall parameters

Time-averaged WSS refers to the tangential frictional stress caused by the action of blood flow on the vessel wall. For pulsatile flow, the time-averaged WSS was calculated by integrating WSS magnitude over a cardiac cycle for each tetrahedral element:

$$WSS = \frac{1}{T}\int_0^T |\vec{\tau}_w|\, dt \quad (1)$$

where $\vec{\tau}$ is the instantaneous WSS vector and T is the duration of the cycle.

To describe the temporal disturbance of intra-aneurysmal flow, OSI, a dimensionless measure of directional changes in WSS, was calculated using the formula reported by He and Ku [1]:

$$OSI = \frac{1}{2}\left[1 - \frac{\left|\int_0^T \vec{\tau}_w dt\right|}{\int_0^T |\vec{\tau}_w|\, dt}\right] \quad (2)$$

Note that $0 \leq OSI < 0.5$, with 0 being completely unidirectional shear and 0.5 being completely oscillatory.

Himburg et al. showed that the residence time of particles near the wall is inversely proportional to a combination of WSS and OSI. RRT prolongation corresponds with low and/or oscillatory WSS [2].

$$RRT = \frac{1}{(1 - 2 \times OSI) \times WSS} = \frac{1}{\frac{1}{T}\left|\int_0^T \vec{\tau}_w\, dt\right|} \quad (3)$$

References

1. Ku DN, Giddens DP, Zarins CK, Glagov S. Pulsatile flow and atherosclerosis in the human carotid bifurcation. Positive correlation between plaque location and low oscillating shear stress. *Arteriosclerosis*. 1985;5:293-302.
2. Himburg HA, Grzybowski DM, Hazel AL, LaMack JA, Li XM, Friedman MH. Spatial comparison between wall shear stress measures and porcine arterial endothelial permeability. *Am J Physiol Heart Circ Physiol.* 2004;286:H1916-22

Supplemental Content 2

Viscosity

Distribution of blood viscosity at the luminal wall of each aneurysm predicted using two rheology models. The Herschel-Bulkley models predicted higher viscosity than the Newtonian viscosity models in AN2, AN5 and AN8 (black arrows).

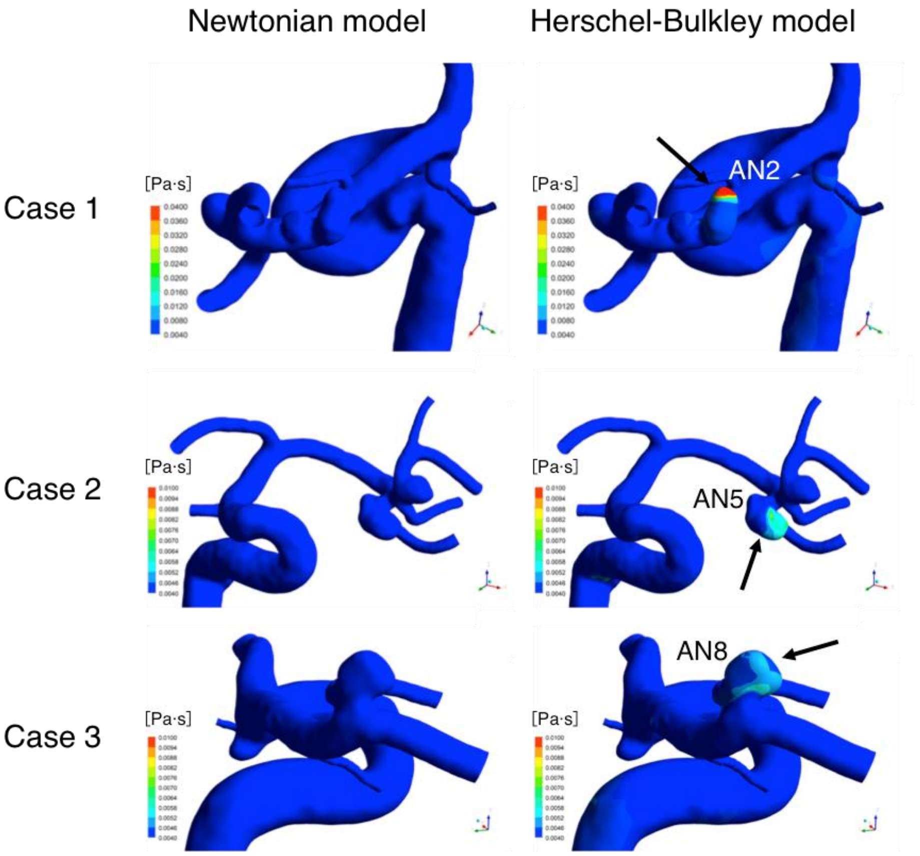

References

1. Rayz VL, Boussel L, Lawton MT, et al. Numerical modeling of the flow in intracranial aneurysms: prediction of regions prone to thrombus formation. *Ann Biomed Eng.* 2008;36:1793-1804.
2. Xiang J, Tremmel M, Kolega J, Levy EI, Natarajan SK, Meng H. Newtonian viscosity model could overestimate wall shear stress in intracranial aneurysm domes and underestimate rupture risk. *J Neurointerv Surg*. 2012;4:351-357
